# A Membrane-Based Electro-Separation Method (MBES) for Sample Clean-Up and Norovirus Concentration

**DOI:** 10.1371/journal.pone.0141484

**Published:** 2015-10-29

**Authors:** Wei Kang, Jennifer L. Cannon

**Affiliations:** Center for Food Safety, The University of Georgia, Griffin, Georgia, United States of America; University of Florida, UNITED STATES

## Abstract

Noroviruses are the leading cause of acute gastroenteritis and foodborne illnesses in the United States. Enhanced methods for detecting noroviruses in food matrices are needed as current methods are complex, labor intensive and insensitive, often resulting in inhibition of downstream molecular detection and inefficient recovery. Membrane-based electro-separation (MBES) is a technique to exchange charged particles through a size-specific dialysis membrane from one solution to another using electric current as the driving force. Norovirus has a net negative surface charge in a neutrally buffered environment, so when placed in an electric field, it moves towards the anode. It can then be separated from the cathodic compartment where the sample is placed and then collected in the anodic compartment for downstream detection. In this study, a MBES-based system was designed, developed and evaluated for concentrating and recovering murine norovirus (MNV-1) from phosphate buffer. As high as 30.8% MNV-1 migrated from the 3.5 ml sample chamber to the 1.5 ml collection chamber across a 1 μm separation membrane when 20 V was applied for 30 min using 20 mM sodium phosphate with 0.01% SDS (pH 7.5) as the electrolyte. In optimization of the method, weak applied voltage (20 V), moderate duration (30 min), and low ionic strength electrolytes with SDS addition were needed to increase virus movement efficacy. The electric field strength of the system was the key factor to enhance virus movement, which could only be improved by shortening the electrodes distance, instead of increasing system applied voltage because of virus stability. This study successfully demonstrated the norovirus mobility in an electric field and migration across a size-specific membrane barrier in sodium phosphate electrolyte. With further modification and validation in food matrixes, a novel, quick, and cost-effective sample clean-up technique might be developed to separate norovirus particles from food matrices by electric force.

## Introduction

Norovirus (NoV) is recognized as the major cause of foodborne illnesses in the U.S. each year, responsible for 58% of foodborne gastroenteritis and 95% of nonbacterial gastroenteritis [1, 2]. The economic impact of norovirus related outbreaks is estimated to be $5.8 billion annually in the U.S. [3]. Norovirus is highly contagious through person-to-person contact and can effect humans of all ages [4]. Indirect norovirus transmission occurs by consumption of fecally contaminated foods or water, or contact with contaminated environmental surfaces [5]. Fresh fruits and vegetables, ready-to-eat deli meats, leafy-green salad mixes, and shellfish are susceptible to norovirus contamination [6]. Oysters can bio-concentrate virus in their tissue when grown in contaminated water. High-level post-handling processing of ready-to-eat deli meats and salad mixes is the primary contamination route of food handlers. Fresh fruits, vegetables, and raw oysters have higher chances of causing norovirus infection because they are often consumed without cooking. The National Outbreak Reporting System (NORS) reported 1,008 norovirus foodborne outbreaks in the U.S. from 2008–2012, wherein 30% were caused by consumption of norovirus contaminated leafy vegetables, 21% by fruits, and 19% by shellfish [7].

To protect public safety, sensitive, fast, and reliable methods to detect norovirus in a variety of foods and drinking water is needed. Such detection systems must also be cost-effective for industry and laboratory uses. Norovirus detection from foods can be divided into two processes, upstream sample preparation and downstream molecular detection. Upstream sample preparation involves virus elution from food matrices and further concentration and purification if needed. Downstream molecular detection involves viral RNA extraction and RT-qPCR detection. Reliable sample preparation requires efficient elution of virus from different kinds of complex food matrices without the co-elution of possible inhibitors for RT-qPCR assays [8]. Elution-concentration is the most common sample clean-up and norovirus concentration method used for a wide range of foods such as carbohydrate-based fresh produce, protein-based read-to-eat foods, and shellfish [9]. However, since this procedure is cumbersome and labor-intensive, inefficient in terms of virus recovery, and potentially introduces inhibitors of downstream molecular detection methods, there is a need to develop better norovirus detection methods from foods [10].

Membrane liquid-phase extraction (MLPE) [11] methods utilize either size- or charge- specific electro-separation membranes and apply an electric field across the membrane to selectively mobilize charged compounds for separation and concentration. MLPE, also known as electrodialysis, is widely used in sample pretreatment in analytical chemistry to extract analytes from complex samples [12–15]. By coupling electro-separation with chromatography or capillary electrophoresis online or offline, an automated detection system can be designed to combine sample pretreatment and analysis for time and cost reduction [16–18]. Utilization of an electric field to separate live cells was first patented using a modification of Gradiflow Technology (Gradipore, Australia), which was a membrane-based electrophoresis method. In that study, negatively charged erythrocytes (7 μm diameter) were mobilized in an electric field across a size-selective membrane (10 μm) and were separated from leukocytes (8–20 μm diameter) in small volume (450 μl) blood samples [19]. Norovirus capsids are 27 nm to 38 nm in diameter and have isoelectric points (pI) of pH 5.5 to 6.5 [20], which means they have a negative surface charge in a neutral or basic pH environment. Similar to red blood cells, they should be able to move in an electric field towards a positive electrode (anode). Therefore, selective separation of norovirus from food samples, with simultaneous separation of inhibitors, may be accomplished with a method using the integration of a pore-size specific membrane barrier and application of electric field.

The objective of this study was to evaluate the feasibility of using a membrane-based electro-separation (MBES) method to evaluate norovirus mobility using an electric field as the sole driving force across a pore-size specific selective separation membrane. This is a proof-of-concept study where an ElectroPrep electrodialysis device (Harvard Apparatus, MA) was adapted for our uses and murine norovirus (MNV-1) was used as the model virus to study this objective. Parameters that effect virus movement in the MBES system were evaluated and its compatibility with elution buffers commonly used in food virology was investigated. The study provides a foundation for designing a detection device to be used for separation and concentration of norovirus from food samples. Future studies should thus validate the method in the context of food matrixes.

## Materials and Methods

### MNV-1 stock preparation

Murine norovirus (MNV-1) was a gift from Dr. Herbert Virgin at Washington University. RAW 264.7 cells for MNV-1 infection were purchased from ATCC® (TIB-71; Manassas, VA). To prepare high titer virus stocks, confluent RAW 264.7 cells in T175 flasks were inoculated with MNV-1 and incubated at 37°C with 5% CO_2_ for 48 hrs. The infected flasks were frozen (-70°C) and thawed (room temperature) for 3 cycles, followed by centrifugation at 2,000 x g for 15 min and vacuum filtration (0.2 μm PES membrane filter) to remove large cell debris and clarify viruses. The filtrate was further concentrated by ultracentrifugation in a Beckman Coulter Ultracentrifuge (XL-80) using a Type 35 rotor at 100,000 x g for 1 hr. The pellet was dissolved in sterile PBS overnight at 4°C to prepare virus stock. Sucrose purification was performed to further concentrate the virus stock. Virus stock was loaded on top of a 30% sucrose solution for centrifugation using a NVT 90 rotor at 100,000 x g for 1 hr at 4°C, and the pellet was dissolved in PBS.

### MNV-1 quantification by plaque assay

MNV-1 virus stock titer was estimated by plaque assay. RAW 264.7 cells were infected with 100 μl of virus stock (~10^7^ pfu/ml) and incubated for 1 hr with gentle rocking manually at 15 min intervals. The surface medium was then aspirated and an agar containing 50:50 of 1% LE agarose (Gold Bio, St. Louis, MO) and 2X minimum essential medium (MEM) was poured over the infected cells. 2X MEM consisted of Cellgro MEM powder (Corning Mediatech, Manassas, VA), 10% Hyclone fetal bovine serum (GE Healthcare, Pittsburgh, PA), 3% HEPES (Lonza), 2% penicillin/streptomycin (Lonza), 2% sodium pyruvate (Corning Mediatech), 2% L-Glutamine (Lonza), 2% non-essential amino acids (Lonza) and 3% sodium bicarbonate (Lonza). The plates were incubated at 37°C with 5% CO_2_ for 48 hrs. PBS containing 3.7% formaldehyde (Acros Organics) was then added to fix the cells on the plates for 2 hrs. The agar layer was removed and the plates were stained with 1% crystal violet (Alfa Aesar, Ward Hill, MA). Visual plaques were counted to calculate the plaque forming units per ml (pfu/ml).

### Membrane-based electro-separation (MBES) device configuration and experimental variables investigated

**Fig 1:**
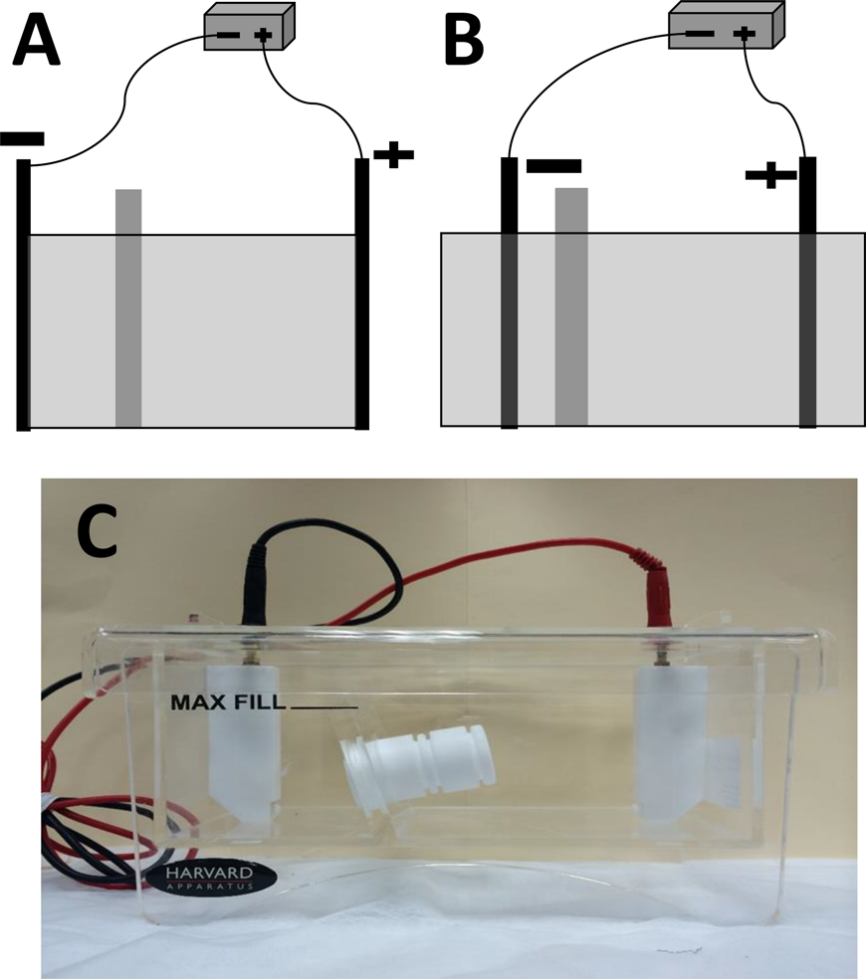
Eletroprep tank used for membrane-based electro-separation (MBES). (A) electrodes originally fixed on the edges of the tank with distance = 18 cm; (B) electrodes reconstructed to the center of the tank with distance = 6 cm; (C) actual picture of the electroprep tank with distance = 18 cm.

An ElectroPrep System including an ElectroPrep tank, two dialyzer chambers (0.5 ml and 1.5 ml), and a union (3.5 ml) were obtained from Harvard Apparatus (Holliston, WA). As shown in Fig 1A and 1C, the original distance between the electrodes of the system was 18 cm. This electrode distance was used initially, but the system was later reconstructed in order to obtain a shorter distance between the electrodes. The electrodes originally fixed on the edges of the electroprep tank were moved to the center of the tank, shortening the distance between the two electrodes to 6 cm (Fig 1B).

**Fig 2:**
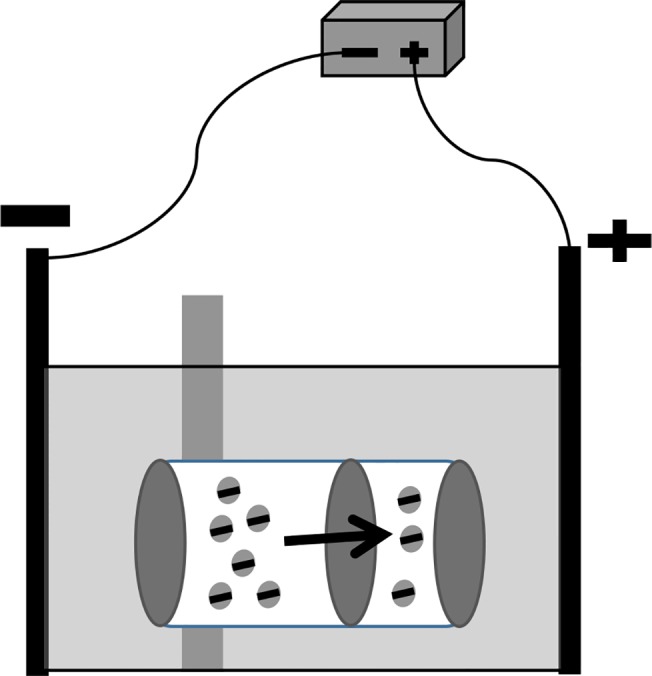
Illustration of viral particle movement in the MBES device.

The union (3.5 ml) was connected to two dialyzer chambers (0.5 ml and 1.5 ml) on each side to create a linked chamber. A pore-size selective polycarbonate membrane (1 μm; SterliTech Corp, Kent, WA), was fitted into the linked chamber and two 300 kDa MWCO cellular acetate restricting membranes (Harvard Apparatus) were flanked on both sides of the linked chambers to create two membrane-separated chambers; one for sample addition (4.0 ml sample chamber) and one for sample collection (1.5 ml collection chamber) (Fig 2). Both chambers were filled with sodium phosphate buffer solutions (pH 7.5; concentration range from 20 mM to 100 mM) with or without the addition of 0.01% SDS or other norovirus elution buffers (PBS, tris-glycine, or TGBE), all with 0.01% SDS addition.

In each experiment, 50 μl of MNV-1 (containing about 10^7^ genomic copies) was added to the sample chamber. The linked chambers were then submerged into the ElectroPrep tank containing 1 L of electrolyte (the same as the buffer solution used to fill the linked chamber), with the sample chamber facing the cathode and the collection chamber facing the anode. A constant voltage potential (range from 20V to 150V) was supplied by a BioRad PowerPac 300 for a period of time (range from 5 min to 60 min) at room temperature to generate an electric field. Samples for which no constant voltage (0V) was applied to the linked chambers were immersed in electrolyte for 30 min as a control for assessing simple diffusion of virus across the selective membrane. At the end of each experimental trial, 300 μl of sample was collected from both the sample and collection chambers and frozen immediately at -70°C until RNA extraction.

For each experiment, electrolyte conductivity and pH were measured by an Orion Star A215 Meter (Thermo Scientific, Waltham, MA). The corresponding current for each applied constant voltage was recorded and the system resistance was calculated using Ohm’s Law:

$$current\;=\;\frac{voltage}{resistant}.$$

Electrolyte and conductivity measurements were repeated a total of six times on different days for experiments using the 20 mM sodium phosphate buffer. For this buffer, the standard deviations for each measured parameter were: initial pH ± 0.03, final pH ± 0.13, initial conductivity ± 0.16, final conductivity ± 0.03, and current ± 1.14. Since these values did not deviate very much from the mean from day to day/experiment to experiment, we chose to only take one measurement for each experiment using the other buffers described in this study.

### RNA extraction

The RNA extraction method used in all the experiments was a modification of Boom’s nucleic acid purification method using an in-house made Guanidine thiocyanate (GuSCN) lysis buffer as the chaotropic agent and silica as binding agent [21]. To make the GuSCN lysis buffer, 60 g guanidine thiocyanate was dissolved in 50 ml 0.5X TE (Tris-EDTA) buffer, followed by addition of 5.5 ml 5 M sodium chloride, 5.5 ml sodium acetate, and 1.1 ml polyadenylic acid potassium salt. Equal volumes of GuSCN lysis buffer and sample (300 μl) were vortexed thoroughly followed by room temperature incubation for 10 min. To determinate the virus input, 50 μl MNV-1 aliquot was mixed with lysis buffer. To precipitate nucleic acid out of solution, a 2X volume of 100% ethanol was added to the mixture and vortexed before transferring to a RNA spin column (Omega Bio-Tek, Norcross, GA) for centrifugation at 14,000 x g for 1 min. The supernatant was discarded and 500 μl of 75% ethanol was added to the column for washing. This was followed by another cycle of centrifugation for 1 min. Then, an additional centrifugation step for drying the column was applied for 1 min after discarding the supernatant. The RNA bound to the column was eluted with 40 μl nuclease-free water (IBI Scientific, Peosta, IA) into a 1.5 ml microcentrifuge tube by centrifugation at the same speed for 1 min.

### Real-time RT-PCR

The quantification of MNV-1 RNA genomic copies was carried out by real-time reverse transcriptase quantitative polymerase chain reaction (RT-qPCR) using a MNV-1 specific probe G54808P (CTA CCC ACC AGA ACC CCT TTG AGA CTC) and primer pair G54763F (TGA TCG TGC CAG CAT CGA) and G54863G (GTT GGG AGG GTC TCT GAGA CAT) [22]. PCR amplification was performed using a Stratagene Mx3005P qPCR System (Aligent Technologies, Santa Clara, CA) using thermal cycle conditions: one cycle of 50°C for 30 min as reverse transcription step; one cycle of 95°C for 15 min as initial PCR activation step; 50 cycles of 95°C for 10 s, 50°C for 30 s, and 72°C for 30 s as denaturation, annealing, and extension steps, respectively. Data collection and analysis was performed using the MxPro software based on a standard curve, consisting of a 10-fold serial dilution of a MNV-1 RNA transcript (see below) ranging from 0 to 10^7^ genome copies per μl. All the samples and controls were tested in duplicate and the PCR efficiency for each standard curve generated ranged from 90% to 110%.

The RNA transcripts were prepared using a MEGA shortscript high yield transcription kit (Ambion, Austin, TX) by *in vitro* transcription of MNV-1. The RNA template for *in vitro* transcription was produced by RT-PCR using MNV-1 pairs G54-T7 (TAA TAC GAC TCA CTA TAC GTC TTG ATC GTG CCA GC) and G54-linker (TAG TAC ATA GTG GAT CCA GCC ATT AGT TGG GAG GGT CTC). The PCR products were transcribed using a MEGAshortscript T7 kit following the manufacturer’s instructions, incubating at 37°C for 4 to 5 hrs, followed by TURBO DNase treatment to clean the transcript. Transcript concentration was measured using a NanoDrop 1000 Spectrophotometer (Thermo Fisher Scientific, Wilmington, DE) and confirmed by RT-qPCR using probe G54808P and primer pairs G54763F and G54863G (described above).

### Data analysis

After each experiment, recovery percentages of MNV-1 from both the sample and collection chambers were calculated by dividing the viral genomic copy number recovered in each chamber by the initial input in the chamber and multiplying by 100%.

$$\%\;\text{recovery in sample chamber}=\frac{genomic\;copies\;detected\;by\;RT-qPCR\;after\;electro-separation\;in\;sample\;chamber\;}{input\;(genomic\;copies)}\;\times 100\%$$

$$\%\;\text{recovery in collection chamber}=\frac{genomic\;copies\;detected\;by\;RT-qPCR\;after\;electro-separation\;in\;collection\;chamber\;}{input\;(genomic\;copies)}\;\times \;100\%$$

### Norovirus mobility in electric field without the presence of membrane

**Fig 3:**
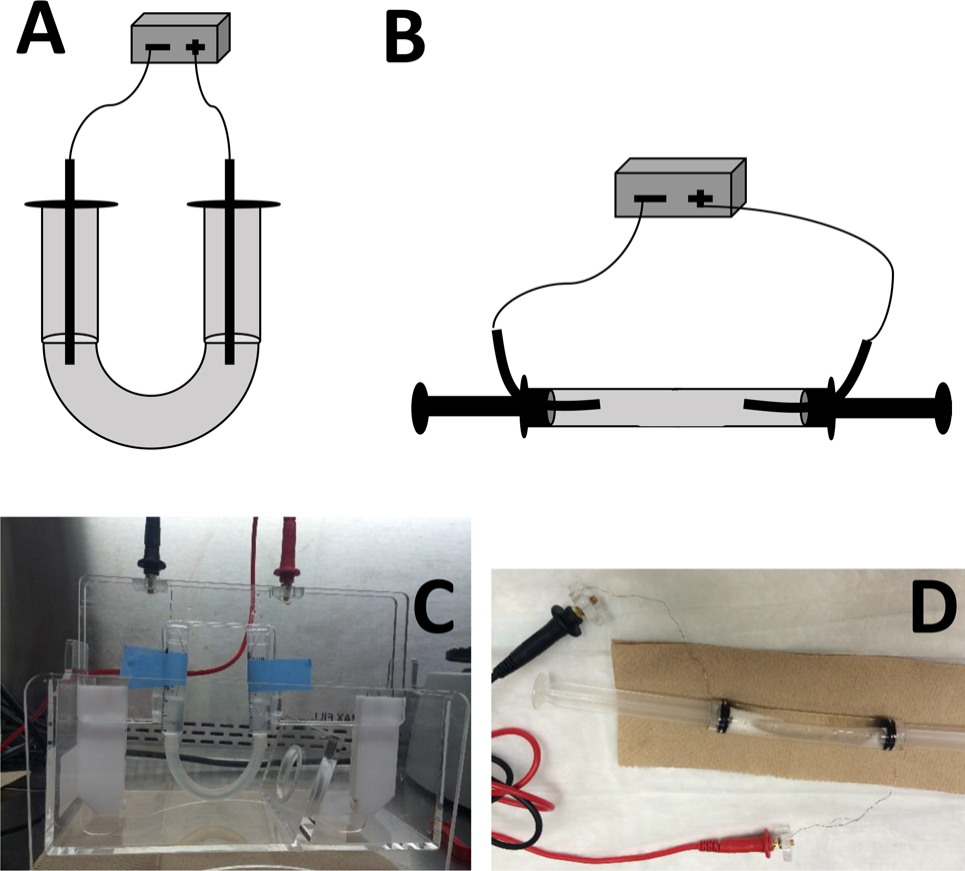
Devices used to investigate viral particles movement in electric field without the presence of membrane barrier. (A) illustration of the U-shape device; (B) illustration of the horizontal device; (C) actual picture of the U-shape device; (D) actual picture of the horizontal device.

In a subset of experiments, a simple device was constructed to evaluate norovirus mobility in electric field without the effect of physical membrane barrier. Two 5 ml syringe barrels (Becton, Dickinson and Company, Franklin Lakes, NJ) were connected with a 6 cm tubing (1/2” ID × 5/8” OD × 1/16” wall Tygon®S3^TM^ E-3603 laboratory tubing; United States Plastic Corp, Lima, OH). Platinum electrode wires connected to the power supply were inserted into the open ends of the syringe barrels to create an electric circuit. In one set of experiments, the device was bent into a U-shape and fixed on a rack (Fig 3A and 3C). Sodium phosphate buffer (500 mM, pH 7.5) with 0.01% SDS was filled into the device followed by 50 μl (7 log genomic copies) of MNV-1 addition to the syringe barrel with negative electrode wire (cathode) inserted. 20V constant voltage (corresponding to 18 mA electric current) was applied to the U-shape device for 30 min. Then the middle section of the tubing was clamped by an office clamp to stop the fluidic flow. After removing the applied electricity, the total sample volumes from the cathode and anode compartments were collected for RNA extraction and RT-qPCR detection. In the second set of experiments, the U-shape device was modified and a horizontal device was constructed. The plungers for the two syringe barrels were inserted to the 6 cm tubing (described above) to prevent liquid flow out of the tubing. Platinum wire, connected to a power supply, was inserted into holes drilled on each plunger (self-sealed with its rubber gasket) to establish an electric circuit (Fig 3B and 3D). Holes were punched in the top of the horizontally placed tubing to allow the passage of gas generated when the electric current was applied. Sodium phosphate buffer with 0.01% SDS (60 mM, pH 7.5) was filled into the tubing along with 50 μl (7 log genomic copies) MNV-1. 40V (corresponding to 30 mA electric current) was applied to the system for 10 min and 30 min, then the middle section of the tubing was clamped and the samples processed as described above. In both sets of experiments, no voltage (0V) was applied for 30 min to serve as the control for assessing virus movement by simple diffusion in the system.

### Statistical analysis

Statistical analysis of experimental data was performed by JMP 12 (SW) (SAS Institute Inc., Cary, NC). Significant differences in % MNV-1 genomic copies recovered from collection chambers impacted by SDS addition to the electrolyte buffer and impacted by electric field strength were analyzed by one-way ANOVA. The means of % MNV-1 genomic copies recovered from collection chambers affected by different electrolyte buffers (PBS, tris-glycine, TGBE, 20 mM sodium phosphate; all with 0.01% SDS addition), different durations, and voltages were compared using student’s t-test for independent samples. Alpha (α) = 0.05 was used to determine significance differences between the means.

## Results and Discussion

### Lessons learned from initial experiments

In initial experiments, to test MNV-1 movement using the MBES system, tris-glycine (pH 7.5) was used as the electrolyte buffer with a 0.6 μm polycarbonate membrane as the separation membrane. Less than 1% of virus was recovered in the collection chamber with applied voltages of 20V-120V for 30 min (data not shown). Then, with the addition of 0.1% sodium dodecyl sulfate (SDS) to the electrolyte buffer (tris-glycine) and substitution with a 1 μm separation membrane, virus recovery in the collection chamber increased to 14.5% with 20V applied voltage for 30 min. The addition of 0.1% SDS to tris-glycine electrolyte increased virus recovery in the collection chamber from below 1% to 14.5%. When another electrolyte (sodium phosphate, concentration ranging from 20 mM to 100 mM, pH 7.5, 0.1% SDS added) was used, the virus recovery in the collection chamber ranged from 5.6% to 19.3% with 20V applied voltage for 30 min. Increased electrolyte concentration seemed to slightly increase virus recovery. Because virus inactivation can occur when too high of a concentration of SDS (0.1%) is used, SDS addition was decreased to 0.01%, which also resulted in a 14.2% virus recovery in the collection chamber when 20V was applied for 30 min using a 20 mM sodium phosphate electrolyte buffer (pH 7.5). The pH of the 20 mM sodium phosphate was increased to 8.5 in an attempt to increase the negative charge of norovirus to see if this would improve virus movement to the sample chamber, but this resulted in similar virus recovery (15.3%) in the collection chamber with the same voltage (20V) and duration (30 min) applied. Although the electrolyte buffer selection seemed to have an impact virus recovery in the collection chamber, increasing the strength and duration of the voltage applied did not seem to increase virus recovery in the collection chamber.

### Improved virus recovery by shortening the distance between electrodes

In 2005, Ogle et al. patented an electrophoresis device (Gradiflow^TM^ Technology) and demonstrated the migration of charged macromolecular solutes through a dialysis membrane [23]. In the report, they described how the distance between electrodes affected the system electric field strength by equation E = V/d (E = electric field strength, V = voltage, d = distance). Therefore, a shorter distance between electrodes will increase the electric field strength of the system when the applied voltage is kept constant. Thus shortening the distance between electrodes may also enhance virus movement across the separation membrane. The electrodes originally fixed in the ElectroPrep tank used in this study were 18 cm apart (d = 18 cm), giving E = 1.11 V/cm when the applied voltage was 20V. After shortening the distance between electrodes to 6 cm (d = 6 cm), E increased to 3.33 V/cm with the same constant voltage (20V) applied.

**Fig 4:**
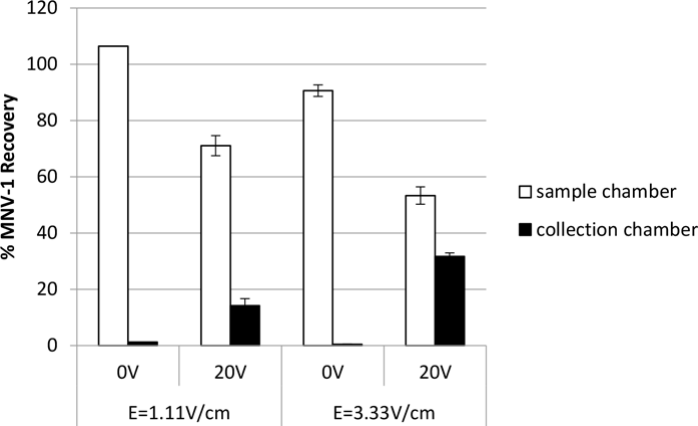
The impact of electric field strength on MNV-1 recovery in the MBES system. Using the system with an electrode distance of 18 cm (E = 1.11V/cm) or 6 cm (E = 3.33V/cm) apart, 7 log genomic copies of MNV-1 were added to the sample chamber prior to voltage application (20V) or without voltage (0V) application for 30 min in a 20 mM sodium phosphate with 0.01% SDS electrolyte buffer. Error bars represent standard deviations, n = 2.

As shown in Fig 4, the recovery of MNV-1 in the collection chamber was less than 1% when no voltage (0V) was applied to the system due to passive diffusion of virus across the membrane (see also Table A in S1 Text). Using a constant voltage of 20V, MNV-1 movement from sample chamber to collection chamber increased significantly (p = 0.0255) when the electric field strength increased, resulting in recovery percentages of 14.2% and 31.7% for the electric field strengths of 1.11 V/cm and 3.33 V/cm, respectively.

### The addition of SDS to the electrolyte buffer improves norovirus recovery

**Fig 5:**
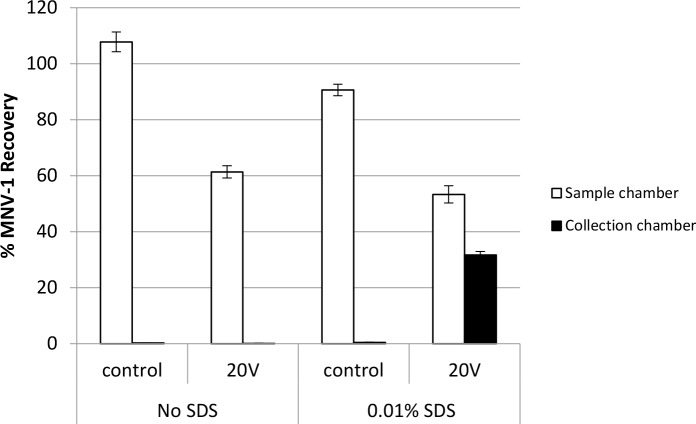
The impact of 0.01% SDS addition to the electrolyte buffer on MNV-1 recovery in the MBES system. Using a 20 mM sodium phosphate electrolyte buffer (pH 7.5) with or without the addition of 0.01% SDS, 7 log genomic copies of MNV-1 were added to the sample chamber prior to voltage application (20 V) or without voltage (0V) application for 30 min. Error bars represent standard deviations of the means, n = 2.

In our preliminary experiments, including SDS in the electrolyte buffer was found to be important for virus recovery from the collection chamber (data not shown). Therefore it was important to demonstrate this phenomenon using the newly designed system having a 6 cm distance between the electrodes. As shown in Fig 5, the addition of 0.01% SDS to the electrolyte buffer improved virus recovery in the collection chamber (see also Table B in S1 Text). With SDS, 31.7% of MNV-1 was recovered from the collection chamber when 20V were applied for 30 min, while 53.3% was recovered in sample chamber. Comparing these results to the MNV-1 recovery results using the electrolyte buffer without SDS, 0% and 61.3% were recovered from collection and sample chambers, respectively. In the control experiments, where no voltage was applied for 30 min, the majority of MNV-1 was recovered in the sample chamber, 90.6% and 107.8% with and without 0.01% SDS, respectively, while 0.4% and 0.2% were recovered from the collection chambers, respectively, due to passive diffusion. The addition of 0.01% SDS to the electrolyte buffer significantly (p = 0.0177) increased the virus movement from sample chamber to collection chamber when 20V were applied for 30 min using the MBES system.

We found that the addition of 0.01% SDS to buffer electrolyte was necessary to enhance viral recovery assisted by an electric field. The MNV-1 genomic copies recovery increased from 0% (without 0.01% SDS) to 31.7% (with 0.01% SDS), as shown in Fig 5. There are two explanations for this phenomenon. First, the addition of detergents such as SDS (also known as sodium lauryl sulfate (SLS)) can reduce surface tension of viral particles, thereby reducing virus absorption to the membrane [24]. Second, the binding of SDS to norovirus particles may have increased the negative surface charge of the particles, thus increasing their movement in the electric field.

When determining particle size of type I poliovirus by syringe filtration in 1968, the addition of 1% SLS to the virus suspension enhanced virus movement through the filter membrane such that 100% of virus particles were recovered in the filtrate using a membrane with a pore-size at least twice as large as the virus. When comparing this to a virus suspension without the addition of SLS, 99% of the virus absorbed to filter membrane. Keesom et al. reported that polycarbonate membranes had a slight negative charge due to a negative zeta potential (-27mV) when the membranes were placed in a buffer with a pH above 6 [25]. Anionic surfactants such as SDS enhance the negative charge of polycarbonate membranes by co-ion surfactant adsorption [25, 26]. SDS could have been bound to the negatively charged polycarbonate membrane used in this experiment, thereby reducing the absorption of MNV-1 to the membrane and leading to an increase in MNV-1 movement across the membrane which would have improved viral recovery in the collection chamber. On the other hand, SDS may have been bound to the surface of viral particles, thereby increasing the particles’ electrostatic repulsion and thus decreasing virus aggregation and their adherence to the membranes. In a study recovering microorganisms from drinking water by ultrafiltration, the addition of the negatively-charged surfactant, sodium pholyphosphate (NaPP), to tap water enhanced recovery of bacteriophage MS2 (108%) and *Salmonella* (49%); when compared to the control samples without NaPP, the recoveries were 51% and 31%, respectively [27]. NaPP added to the water samples was thought to bind to the microorganisms, which decreased the surface zeta potentials of the microorganisms, making their surface charge more negative [28]. SDS, also a strong negatively-charged surfactant, may have the same function as NaPP in this study. The sum of virus recovery in the sample and collection chambers was about 60% and 80% without and with SDS added to electrolyte when 20V was applied, respectively (Fig 5). Therefore, it is likely that without the binding of SDS to both membrane and viral particles, virus adherence or absorption to the membranes was increased.

Although norovirus viral particles have a negative charge under neutral or basic aquatic environments [20], the surface charge appeared to be too weak to allow particle movement in such a weak electric field (20V). Similar to the function of SDS in SDS-PAGE (sodium dodecyl sulfate polyacrylamide gel electrophoresis) [29], the hydrophobic binding of the anionic detergent, SDS, to the surface of viral particles likely increased the overall negative viral surface charge, thereby strengthening the viral particle movement towards the anode (positive electrode).

It is very likely that both hypotheses of the function of SDS contributed to the improvement of virus recovery in the collection chamber. Based on the findings in Fig 5, 0.01% SDS was included in the electrolyte buffer for all subsequent experiments optimizing our MBES system.

### Electrolyte type and concentration is important for virus movement and survival in the system

With the modification of the Ogle et al. (2005) electrophoresis device, a fixed boundary electrophoresis method to separate living cells in a membrane-based electro-separation methodology was patented under Gradiflow^TM^ Technology [19]. They demonstrated that 10^5^ to 10^10^ cells/ml of erythrocytes (7 μm in diameter) (~80%) migrated through a 10 μm polycarbonate membrane in 2 min with an electric field of 50V/cm using a broad variety of electrolyte buffers such as Bis-Tris, HEPES, glucose, sucrose, and phosphate buffer salts (all pH 7.4, conductivity 4 mS/cm) in small volume (anodic and cathodic chambers were both 420 μl). They also described how the buffer concentration and conductivity, electric field strength, and applied voltage all affected cell migration, but the selection of buffer did not seem to matter. As described in our preliminary studies, the type of buffer used in MBES system did appear to impact virus movement; therefore, we further investigated if the buffer type and concentration would affect norovirus migration across the separation membrane under the mild electric field strengths used in our system.

**Fig 6:**
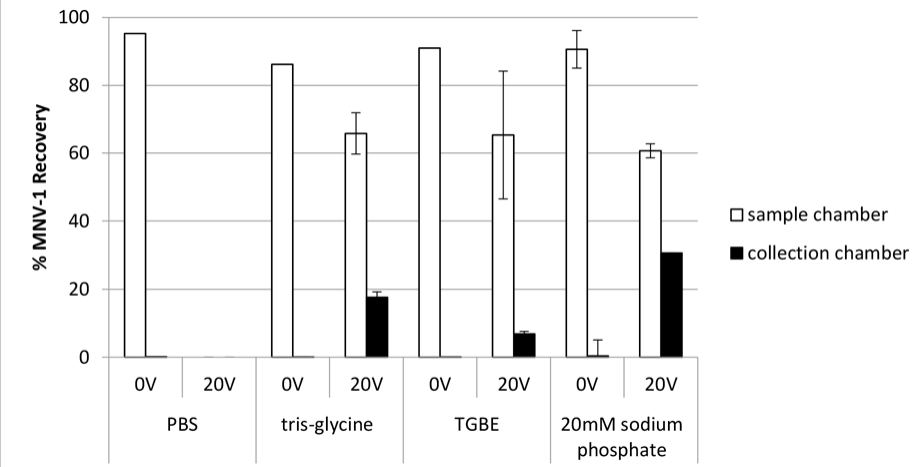
The impact of buffer selection on MNV-1 recovery in the MBES system. Using PBS (pH 7.4) with 0.01% SDS, tris-glycine (pH 8.3) with 0.01% SDS, TGBE (pH 8.8) with 0.01% SDS, or 20 mM sodium phosphate (pH 7.5) with 0.01% SDS as electrolyte buffers, 7 log genomic copies of MNV-1 were added to the sample chamber prior to voltage application (20V) and no voltage (0V) application for 30 min. Error bars represent standard deviations, n = 2.

PBS, TGBE [30, 31], and Tris-glycine [32, 33] are common elution buffers used to extract and recover norovirus from the surfaces of different foods or other environmental matrices. Table 1 indicates the pH and buffer conductivity measurements of these elution buffers. A wide range of conductivities (0.7–13.3 mS/cm) are represented by these buffers. However, we also wanted to include a buffer that had a conductivity and pH similar to that recommended in Rylatt and Leong’s patent (buffer pH 7.4 and conductivity 4 mS/cm) [19]. Therefore, we selected a 20 mM sodium phosphate buffer having a conductivity of 3.5 mS/cm and pH 7.4. As shown in Fig 6, when no voltage was applied for 30 min, most of the virus was recovered in the sample chamber (range from 86.1% to 95.3%) for all electrolyte buffers used (PBS, tris-glycine, and 20 mM sodium phosphate, all with 0.01% SDS), while less than 1% virus was recovered in the collection chamber (see also Table C in S1 Text). After applying 20V to the MBES system (electrodes distance 6 cm) for 30 min using each electrolyte buffer with a 7 log genomic copies/ml MNV-1 input, virus recoveries in the collection chamber differed (p < 0.0001) for each buffer (Fig 6). No virus was recovered in sample or collection chambers when PBS was used. Respective sample and collection chamber MNV-1 recovery rates were 65.8% and 17.7% with tris-glycine, 65.3% and 6.9% with TGBE, and 60.7% and 30.8% with 20 mM sodium phosphate.

**Table 1:** pH, conductivity, and electrical current measurements and calculations for common virus elution buffers containing 0.01% SDS.

Electrolyte	Initial pH	Final pH	Initial Conduc-tivity (ms/cm)	Final Conduc-tivity (ms/cm)	Current (mA)*	System resistance (V/mA)**	Current density (A/m^2^)	Electric field strength
**20 mM sodium phosphate**	7.5 (±0.03)	7.5 (±0.13)	3.5 (±0.16)	3.5 (±0.03)	26 (±1.14)	0.8	53.1	15.2
**Tris-glycine**	8.3	8.3	0.7	0.7	4.0	2	8.2	11.7
**TGBE**	8.8	8.8	2.2	2.2	12.5	1.6	25.5	11.6
**PBS**	7.4	3.0 (anode compartment) 11.3 (cathode compartment)	13.3	13.3	92	0.2	187.7	14.1

*20V constant voltage was applied for 30 min in the MBES system.

**System resistance was calculated by Ohm’s law R = V/I where current (I) was recorded during the voltage (20V) application. Current density was calculated by the analogous Ohm’s Law equation, electric field strength (E) = current density (J) / conductivity (σ), where current density (J) = current (A) / membrane area (m^2^) (the membrane area in our system was 4.9 X 10–4 m^2^). The electric field strength was calculated by equation E = V/d (E = electric field strength, V-voltage, d = distance).

For tris-glycine and 20 mM sodium phosphate buffers, the sum of the percent recovery values for the sample and collection chambers totaled approximately 90%. However, this was not the case for TGBE and PBS.

With TGBE, the sum of virus recoveries in the sample chamber and collection chambers was only 72.2% with a large standard deviation for the sample chamber recovery results (65.3% ± 18.8) when 20V was applied. Likely due to the complexity of TGBE, components of this buffer could have interfered with virus movement across the membrane when voltage was applied. No virus was recovered from either the sample chamber or collection chamber when PBS was used as the electrolyte buffer. After applying 20V to the system for 30 min, the pH and conductivity of each buffer remained stable, with the exception of the PBS buffer (Table 1). The pH of the PBS electrolyte in the anode compartment decreased to 3.0 and the pH in the cathode compartment increased to 11.3 after the 20V application for 30 min. This pH change was likely due to the electrolysis of sodium chloride contained in PBS. Hydrolysis caused hydroxide and hydrogen ion formation and a migration of these ions toward the cathode and anode, respectively, resulting in pH changes. Viral capsids and subsequently viral RNA in the sample chamber might have degraded due to the pH increase to 11.3 using PBS. Although a low pH of 3 should not cause MNV-1 inactivation [34], free chlorine generation due to the dissociation of sodium and chloride ions of NaCl in PBS could have caused virus inactivation. Measuring free chlorine levels in this compartment was attempted using the DPD-FEAS (ferrous ethylenediammonium sulfate) titration method (HACH company, Loveland, CO), but free chlorine production could not be detected. It is possible that the volume of electrolyte (1 L) was too diluted for accurate chlorine detection (detection range of DPD-FEAS method was 0–3.00 mg/L free chlorine production in a 25 ml sample volume) or the free chlorine could have been rapidly consumed by the virus before it could be measured.

**Fig 7:**
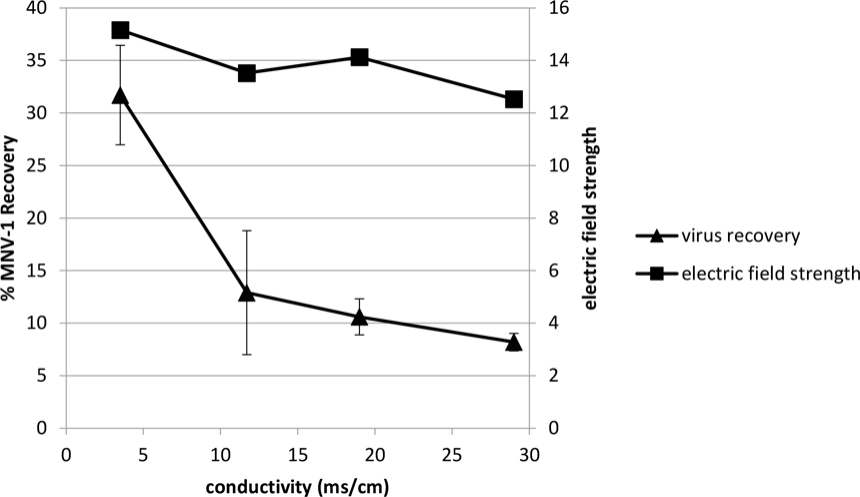
Relationship between phosphate buffer electrolyte conductivity and its associated electric field strength to virus recovery in the collection chamber. Using 20 mM, 100 mM, 200 mM, or 300 mM sodium phosphate (all with 0.01% SDS, pH 7.5) as the electrolyte buffer, 7 log genomic copies of MNV-1 were added to the sample chamber prior to voltage application (20V) and no voltage (0V) application for 30 min. Error bars represent standard deviations, n = 2.

As our preliminary findings suggested, that electrolyte buffer conductivity may also impact virus recovery, we examined different concentrations of phosphate buffer using the MBES system. According to the equation analogous to Ohm’s Law, electric field strength (E) = current density (J) / conductivity (σ), where current density (J) = current (A) / membrane area (m^2^) (the membrane area in our system was 4.9 X 10^−4^ m^2^). Increasing the concentration of phosphate buffer in the system did increase the current density, but because of the larger increase of buffer conductivity, the net electric field strength decreased slightly as the electrolyte buffer concentration increased (Table 2). Interestingly, this slight decrease of the electric field strength for increasing concentrations of phosphate buffer in the system resulted in a substantial decrease of virus recovery from 31.7% to 8.2% (Fig 7; Table D in S1 Text). The highest virus recovery in the collection chamber was achieved when 20 mM sodium phosphate electrolyte (conductivity 3.5 mS/cm) was used, while increasing the electrolyte concentration in an attempt to increase conductivity deceased virus recovery. The optimized electrolyte conductivity (3.5 mS/cm) in this study was similar to Rylatt and Leong’s patent [19], in which the optimized electrolyte conductivity was 4.0 mS/cm. Comparing the conductivity of different concentrations of sodium phosphate and other buffers tested (Tables 1 and 3), a relatively low electrolyte conductivity was needed to increase overall virus movement in a weak electric field. Insufficient electric field was generated to mobilize virus particles when the conductivity was too low, while the net electric field strength started to decrease if conductivity was too high.

**Table 2:** pH, conductivity, and electrical current measurements and calculations for common virus elution buffers containing 0.01% SDS.

Electrolyte	Initial pH	Final pH	Initial Conduc-tivity (ms/cm)	Final Conduc-tivity (ms/cm)	Current (mA)*	System resistance (V/mA)**	Current density (A/m^2^)	Electric field strength
**20 mM sodium phosphate**	7.5 (±0.03)	7.5 (±0.13)	3.5 (±0.16)	3.5 (±0.03)	26 (±1.14)	0.8	53.1	15.2
**Tris-glycine**	8.3	8.3	0.7	0.7	4.0	2	8.2	11.7
**TGBE**	8.8	8.8	2.2	2.2	12.5	1.6	25.5	11.6
**PBS**	7.4	3.0 (anode compartment)11.3 (cathode compartment)	13.3	13.3	92	0.2	187.7	14.1

*20V constant voltage was applied for 30 min in the MBES system.

**System resistance was calculated by Ohm’s law R = V/I where current (I) was recorded during the voltage (20V) application. Current density was calculated by the analogous Ohm’s Law equation, electric field strength (E) = current density (J) / conductivity (σ), where current density (J) = current (A) / membrane area (m^2^) (the membrane area in our system was 4.9 X 10–4 m^2^). The electric field strength was calculated by equation E = V/d (E = electric field strength, V-voltage, d = distance).

**Table 3:** pH, conductivity, and electrical current measurements and calculations for sodium phosphate buffers of different concentrations containing 0.01% SDS.

Concen-tration	Initial pH	Final pH	Initial Conduc-tivity (ms/cm)	Final Conduc-tivity (ms/cm)	Current (mA)*	System resistance (V/mA)**	Current density (A/m^2^)	Electric field strength
**20 mM**	7.5 (±0.03)	7.5 (±0.13)	3.5 (±0.16)	3.5 (±0.03)	26 (±1.14)	0.78	53.1	15.2
**100 mM**	7.5	7.5	11.7	11.7	77.5	0.3	158.2	13.5
**200 mM**	7.5	7.5	19.0	19.0	131.5	0.2	268.4	14.1
**300 mM**	7.5	7.5	29.0	29.0	178	0.1	363.3	12.5

*20V constant voltage was applied for 30 min in the MBES system.

** System resistance was calculated by Ohm’s law R = V/I where current (I) was recorded during the voltage (20V) application. Current density was calculated by the analogous Ohm’s Law equation, electric field strength (E) = current density (J) / conductivity (σ), where current density (J) = current (A) / membrane area (m^2^) (the membrane area in our system was 4.9 X 10–4 m^2^). The electric field strength was calculated by equation E = V/d (E = electric field strength, V-voltage, d = distance).

### The impact of the duration of electric field application on norovirus recovery and stability

**Fig 8:**
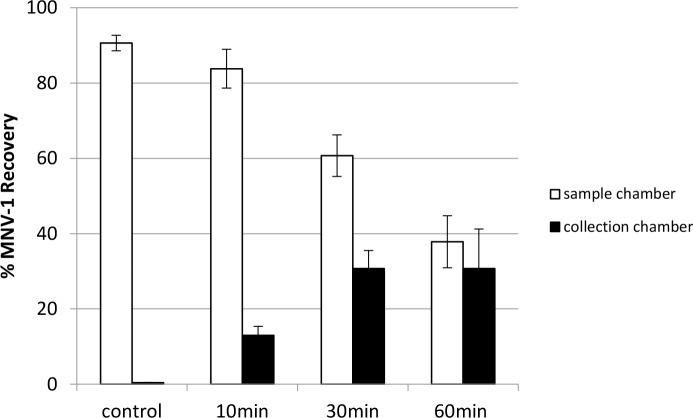
The impact of duration of the applied voltage on MNV-1 recovery in the MBES system. A constant voltage (20V) was applied in the MBES system for 10 min, 30 min, and 60 min or no voltage (0V) for 30 min (control) with 7 log genomic copies of MNV-1 added to the sample chamber prior to voltage application using 20 mM sodium phosphate with 0.01% SDS as the electrolyte buffer. Error bars represent standard deviations, n = 2.

From Fig 8, the recovery of MNV-1 from the collection chamber increased from 13.0% (after 10 min) to 30.8% (after 30 min) when 20V was applied; while MNV-1 recovered from the sample chamber decreased from 83.8% (after 10 min) to 60.7% (after 30 min) (see also Table E in S1 Text). These results showed that as electro-separation duration increased from 10 min to 30 min, more MNV-1 moved from sample chamber to collection chamber, but the result was not statistically significant (p = 0.0992). However, when duration increased from 30 min to 60 min, MNV-1 recovery in the collection chamber remained identical (p = 0.9910), 30.8% (30 min) and 30.7% (60 min), but MNV-1 recovery in the sample chamber decreased from 60.7% (30 min) to 37.9% (60 min).

In a study utilizing stagnant electrodialysis to separate negatively charged inositol with a size-selective membrane [18], inositol phosphate enrichment increased when the electrodialysis time increased from 1 min to 5 min with 600V applied. A non-linear curve of time vs. target enrichment could be plotted. As time increased to 8 min and 10 min, no more enrichment was observed. The results in Buscher’s study were similar to our study in that as duration increased, the recovery increased until a point where increases in virus recovery could no longer be achieved. Gas bubble formation around the electrodes was reported in Buscher’s study and was also observed in the current study. Bubble formation for a long duration may alter and mitigate the electric field strength provided by the same constant voltage due to electrical resistance build-up and current deprivation [18].

Besides the possibility of electric current deprivation causing an increase of electrical resistance [18], viral particles may destabilize with prolonged electric application, causing RNA exposure and degradation by innate RNases present in the electrolyte, since RNase-free buffers were not used in this study. This was likely the reason for the sharp decrease in MNV-1 recovery from the sample chamber after the 60 min duration. The sum of MNV-1 genomic copy recovery rates in the sample chamber (37.9%) and in the collection chamber (30.7%) after 60 min of applied voltage was 68.6%, meaning 31.4% MNV-1 genomic copies were lost due to RNA degradation (Fig 8).

### Moderate applied electric field strength is important to norovirus recovery and stability

**Fig 9:**
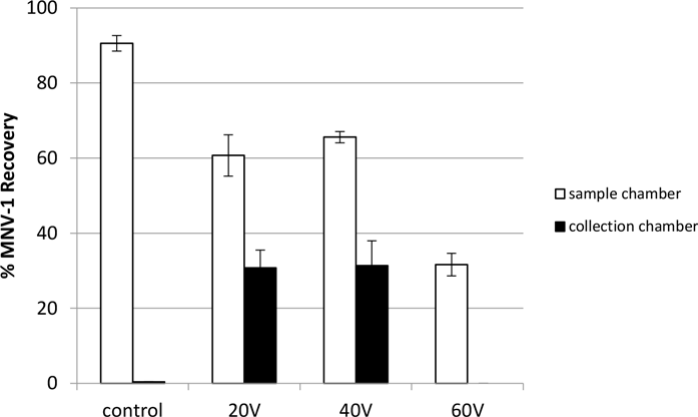
The impact of applied voltage strength on MNV—1 recovery using the MBES system. Applying a constant voltage of 20V, 40V, or 60V and no voltage (control) to the system for 30 min, 7 log genomic copies of MNV-1 were added to the sample chamber prior to voltage application using 20 mM sodium phosphate with 0.01% as the electrolyte buffer. Error bars represent standard deviations, n = 2.

MNV-1 recovery rates in the collection chambers were 30.8% and 31.3% when the applied voltages were 20V and 40V, respectively; while MNV-1 recovery in sample chambers were also similar, 60.7% and 65.5%, respectively. However, when the applied voltage increased to 60V, no MNV-1 was detected in the collection chamber and only 31.6% was recovered in the sample chamber. Almost 70% of MNV-1 was lost after 60V was applied for 30 min (Fig 9). Fig 9 results indicated that when a high voltage is applied for prolonged durations, this could cause viral destabilization and RNA degradation (see also Table F in S1 Text).

**Fig 10:**
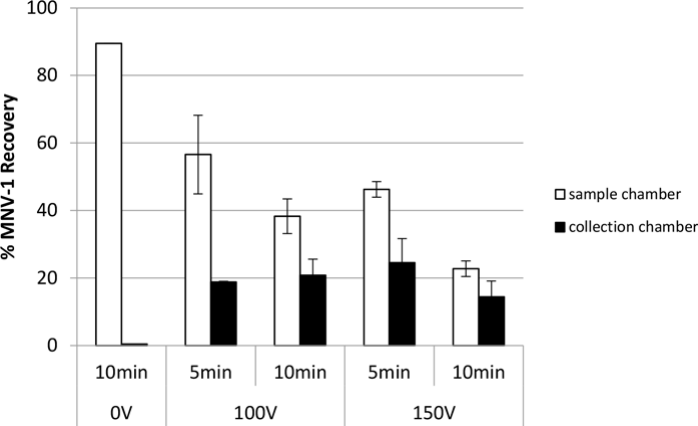
The impact of high applied voltage for a short duration on MNV-1 recovery using the MBES system. Constant voltages of 100V or 150V were applied to the system for 5 min or 10 min and no voltage for 10 min (control). 7 log genomic copies of MNV-1 were added to the sample chamber prior to voltage application using 20 mM sodium phosphate with 0.01% SDS as the electrolyte buffer. Error bars represent standard deviations, n = 2.

When high voltage was applied for short durations in the MBES system, as shown in Fig 10, 18.8% and 24.5% of MNV-1 was recovered in the collection chamber when 100V and 150V was applied for 5 min, respectively (see also Table G in S1 Text). When the applied voltage duration was increased to 10 min, no increase in the collection chamber recovery rates of MNV-1 were observed, but a significant MNV-1 recovery loss (p = 0.0092) in the sample chamber was observed when 150V was applied. The sum of MNV-1 recovery percentages from the sample and collection chambers was 75.4% and 59.1% when 100V was applied for 5 min and 10 min (p = 0.2940), respectively; while 70.8% and 37.2% was observed when 150V was applied for 5 min and 10 min (p = 0.0688), respectively.

In the inositol phosphate separation study [18], an increase in applied voltage from 150V to 600V increased inositol migration from the donor phase to receptor phase through a 30,000 MWCO membrane in 20 seconds, but voltage higher than 600V did not increase inositol phosphate migration due to intense bubble formation near the electrodes and thus destabilization of the electric field. Issaq et al., discussed the relationship between the applied voltage and a compound’s mobility [35] using dansylalanine and mesiyloxide to confirm their hypothesis. An increase in applied voltage caused heat formation near the electrodes, leading to uneven electrolyte temperature increases. Partial temperature increases can cause an increase in buffer conductivity but also a decrease in buffer density and viscosity. The combination of all these effects could result in an increase in solute mobility and thus improve recovery. In a more advanced design based on electric field separation, Balchen et al. extracted angiotensin peptides from human plasmas by electro-membrane extraction (EME) through a supported liquid membrane (SLM) [36]. Angiotensin recovery increased to 30% when voltage increased from 5V to 15V, but no recovery increase was observed when the voltage increased from 15V to 20V. In this case, increasing the applied voltage initially favored angiotensin migration because of the higher electric current generated, but increasing the voltage too much caused electrolyte electrolysis near the electrodes. Thus, an unstable electrical system formed because of bubbles and heat generation. This phenomenon was also observed when basic drug substances were extracted from human plasma and human urine by EME [37].

In capillary electrophoresis (CE), extremely high voltages (usually measured in kilo-voltage (kV)) are used to mobilize biological particles (viruses, bacteria, and eukaryotic cells). Differences in particle sizes, particle surface amino acid residues, and charged sugars can contribute to the different particle mobility across a capillary when voltage potential is applied [38]. A UV or fluorescence detector is connected to the end of the capillary to measure the absorbance of viral capsids or residual viral genomes and impurities that pass through [39]. When determining norovirus VLPs (virus-like particles) isoelectric point, a whole-column UV absorption imaging detector was coupled with a capillary to separate VLPs by 3 kV dc voltage [20]. In such a system, both intact and damaged VLPs would be detected. RT-PCR is usually not used to detect viruses separated by CE due to the likely disruption of intact viruses by high voltage potential. This is the reason why voltages as high as those used in CE technique were not implemented by this MBES study.

### Restriction membranes separating the electrodes from norovirus in the sample and collection chambers is necessary to avoid virus inactivation

When measuring the system resistance with an applied voltage of 20V using the 20 mM sodium phosphate with 0.01% SDS (pH 7.5) as the electrolyte buffer, the system resistance increased respectively from 385Ω to 769Ω without and with the presence of the restriction and separation membranes in the device. The increased resistance also caused a decrease of electric current from 52 mA to 26 mA (voltage = current X resistance), which might affect virus movement causing low virus mobility, and thus a decrease in virus recovery in the collection chamber. To decrease the resistance of the system in an attempt to increase virus mobility, the system was re-designed to remove all membranes. In the first re-design, a U-shaped device was constructed (Fig 3A and 3C). However, due to the vertical nature of the fluid movement in the device and the narrow connection between the syringe barrels and tubing, electron flow was decreased, and thus no electric current was established using the 20 mM sodium phosphate (with 0.01% SDS) buffer electrolyte with 20V applied to the U-shaped device. In an attempt to compensate for this unexpected increase in system resistance, a high concentration of electrolyte buffer (500 mM sodium phosphate) was used to obtain a sufficient electric circuit. When a constant current of 20V was applied, 23 mA electric current was recorded, which was similar to the MBES system’s electric current (26 mA) when using the 20mM sodium phosphate (with 0.01% SDS) electrolyte. However, when 500 mM sodium phosphate (with 0.01% SDS) was used, a precipitate was formed after the samples underwent the normal freeze-thaw cycle prior to the RNA extraction step. No viral RNA genomic copies were detected in either the sample or collection chambers using this system, possibly due to of the presence of the precipitate that clogged the membrane of the spin column used during the RNA extraction step.

**Fig 11:**
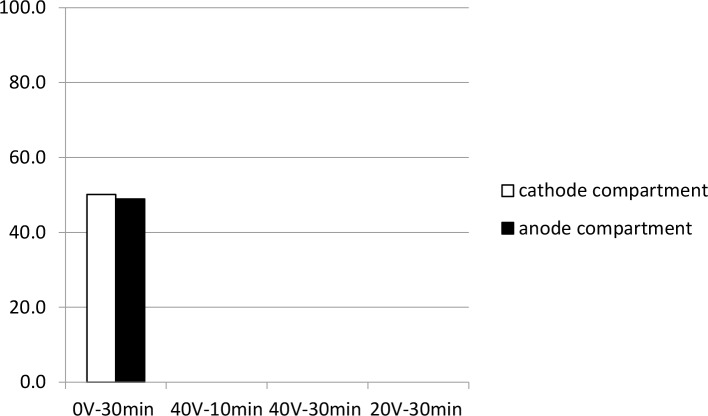
MNV-1 recovery using a horizontal electro-separation device without the addition of membrane barriers for restriction or separation. Using a horizontal device (6 cm length) filled with 60 mM sodium phosphate buffer (pH 7.5) with 0. 01% SDS, 7 log genomic copies of MNV-1 were added to the device prior to voltage application (40V for 10 min, 40V for 30 min, and 20V for 30 min) or no voltage (0V) application for 30 min, (n = 1).

To cope with high system resistance and low electric current due to insufficient electrons flowing in the U-shaped vertical device, the system was again re-designed in a horizontal format (Fig 3B and 3D). As shown in Fig 11, when 0V electrical potential was applied, 50% and 48% MNV-1 was recovered in the cathode and anode compartments, respectively, due to passive diffusion in the tubing (see also Table H in S1 Text). When 20V was applied for either 10 min or 30 min, no RNA was detected in either the cathode or anode compartments.

Without the shielding of the electrodes with a restriction membrane barrier, it is likely that the negatively charged virus was attracted to the anode surface by electrosorption. This would have been followed by direct electron transfer caused by oxidoreduction of the electrolysis buffer, resulting in instant virus inactivation. This phenomenon is similar to the inactivation of *E*. *coli* by electrochemical disinfection using a Pt (platinum) anode and a chlorine-free electrolyte [40]. In Rylatt and Leong’s patent, physical membrane barriers (10 or 20 kDa CTA membrane) were used to prevent the convection of electrolyte between the electrode compartment and sample compartment as well as from the direct contact of the cells to the electrodes [19]. A physical membrane (either pore-selective membrane or ion-exchange membrane) is thus needed to separate the virus sample from direct contact with the electrode surface, which can cause virus inactivation.

## Conclusions

This study is the first to illustrate the possibility of mobilizing viruses, and in particular, noroviruses, by electric force across a pore-size selective separation membrane in order to achieve their separation, concentration and purification. A MBES method for recovering murine norovirus from phosphate buffer was successfully designed, developed, and evaluated in this proof-of-concept study. In a subset of experiments, as high as 30.8% of MNV-1 migrated from sample chamber to collection chamber across a 1 μm separation membrane, when 20 mM sodium phosphate buffer with 0.01% SDS was used with 20V applied for 30 min. Of all variables tested, this set of parameters yielded the highest virus recovery percentage, implying that weak voltage, moderate duration, and low ionic strength electrolytes are optimal for norovirus movement across an electric field without disrupting the stability of the virus particles. Since the negatively charged surface of norovirus is not strong enough to be mobilized in a weak electric field, SDS addition to electrolyte is essential to increase norovirus mobility and recovery. The electric field strength of the system is a key factor to enhance norovirus mobility in an electric field, but from this study it was determined that this can only be achieved by decreasing the distance between the electrodes instead of increasing applied voltage because the virus is not stable at high voltages as demonstrated by RNA degradation. From this study it was also found that a horizontal electric system design is important to ensure the smooth flow of electrons and decrease the system resistance. Last but not least, although it was found that the integration of restriction membranes in the system increases system resistance, a membrane barrier separating the electrode from the collection chambers is essential to avoid virus inactivation by oxidoreduction when in contact with positive electrode (anode).

This study suggests that in future designs, either increasing the separation membrane surface area, decreasing the distance between electrodes, or both can help to improve norovirus recovery beyond what was observed in this study. To accommodate larger sample input volumes, a continuous input feeding design could be explored with this system. MNV-1 infectivity was not evaluated using the MBES system because the virus concentration in each chamber was too low to be detected by plaque assay. Future studies should include a virus concentration step prior to analysis of virus infectivity in order to evaluate whether or not the system could be used to detect infectious virus.

In conclusion, this study was the first to explore the movement of viruses, and in particular, noroviruses, in an electric field as a part of a sample clean-up and preparation method for norovirus detection. With further modifications of the system and optimization of its parameters, the membrane-based electro-separation method may provide a novel, quick, easy, and cost-effective method for norovirus detection. Furthermore, if improved recovery percentages can be achieved with further optimization, it will be necessary to validate the promising method in the context of food matrixes.

## Supporting Information

S1 Text Data supported Figs 4–11 are listed in Tables A-G. (DOCX)
