# Supplementary material for: A Membrane-Based Electro-Separation Method (MBES) for Sample Clean-Up and Norovirus Concentration
Source: PLoS One. 2015 Oct 29;10(10):e0141484. doi: 10.1371/journal.pone.0141484 (PMC4625962; doi:10.1371/journal.pone.0141484)
Supplement: S1 Text — (DOCX) [file pone.0141484.s001.docx]

**Table A. Impact of electric field strength on MNV-1 recovery in MBES system.** Values are average of duplication experiments (n=2).

| **Electric field strength** | | **E=1.11V/cm** | | **E=3.33V/cm** | |
| --- | --- | --- | --- | --- | --- |
| **Voltage** |  | **0V** | **20V** | **0V** | **20V** |
| **% recovery** | sample chamber | 106.4 | 71.0 | 90.6 | 53.3 |
|  | collection chamber | 1.2 | 14.2 | 0.4 | 31.7 |
| **Standard deviation** | sample chamber | 0 | 3.6 | 2.1 | 3.1 |
|  | collection chamber | 0 | 2.5 | 0.1 | 1.2 |

**Table B. The impact of % SDS addition to the electrolyte buffer on MNV-1 recovery in the MEBS system.** Values are average of duplication experiments (n=2).

| **SDS addition** | | **0.01% SDS** | | **No SDS** | |
| --- | --- | --- | --- | --- | --- |
| **Voltage** |  | **0V** | **20V** | **0V** | **20V** |
| **% recovery** | Sample chamber | 90.6 | 53.3 | 107.8 | 61.3 |
|  | Collection chamber | 0.4 | 31.7 | 0.2 | 0.0 |
| **Standard deviation** | Sample chamber | 2.1 | 3.1 | 3.5 | 2.2 |
|  | Collection chamber | 0.1 | 1.2 | 0 | 0.1 |

**Table C: The impact of buffer selection on MNV-1 recovery in the MEBS system.** Values are average of duplication experiments (n=2).

| **Electrolyte** | | **PBS** | | **Tris-glycine** | | **TGBE** | | **20 mM sodium phosphate** | |
| --- | --- | --- | --- | --- | --- | --- | --- | --- | --- |
| **Voltage** |  | **0V** | **20V** | **0V** | **20V** | **0V** | **20V** | **0V** | **20V** |
| **% recovery** | sample chamber | 95.3 | 0.0 | 86.1 | 65.8 | 91.0 | 65.3 | 90.58 | 60.71 |
|  | collection chamber | 0.2 | 0.0 | 0.1 | 17.7 | 0.0 | 6.9 | 0.42 | 30.75 |
| **Standard deviation** | sample chamber | 0.0 | 0.0 | 0.0 | 6.1 | 0.0 | 18.8 | 5.53 | 2.06 |
|  | collection chamber | 0.0 | 0.0 | 0.0 | 1.6 | 0.0 | 0.7 | 4.73 | 0.06 |

**Table D. Relationship between phosphate buffer electrolyte conductivity and its associated electric field strength to virus recovery in the collection chamber.** Values are average of duplication experiments (n=2).

| **Sodium phosphonate concentration** | **Conductivity** | **Electric field strength** | **Virus recovery in collection chamber** | **Standard deviation for virus recovery in collection chamber** |
| --- | --- | --- | --- | --- |
| 20.0 | 3.5 | 15.2 | 31.7 | 4.7 |
| 100.0 | 11.7 | 13.5 | 12.9 | 5.9 |
| 200.0 | 19.0 | 14.1 | 10.6 | 1.7 |
| 300.0 | 29.0 | 12.5 | 8.2 | 0.8 |

**Table E. The impact of duration of the applied voltage on MNV-1 recovery in the MEBS system.** Values are average of duplication experiments (n=2).

| **Duration** | | **control** | **10 min** | **30 min** | **60 min** |
| --- | --- | --- | --- | --- | --- |
| **% recovery** | sample chamber | 90.6 | 83.76 | 60.7 | 37.9 |
|  | collection chamber | 0.4 | 13.02 | 30.8 | 30.7 |
| **Standard deviation** | sample chamber | 2.1 | 5.1 | 5.5 | 6.9 |
|  | collection chamber | 0.1 | 2.4 | 4.7 | 10.5 |

**Table F. The impact of applied voltage strength on MNV-1 recovery in the MEBS system.** Values are average of duplication experiments (n=2).

| **voltage** | | **0V** | **20V** | **40V** | **60V** |
| --- | --- | --- | --- | --- | --- |
| **% recovery** | sample chamber | 90.6 | 60.71 | 65.6 | 31.6 |
|  | collection chamber | 0.4 | 30.75 | 31.3 | 0.0 |
| **Standard deviation** | sample chamber | 2.1 | 5.5 | 1.5 | 3.0 |
|  | collection chamber | 0.1 | 4.7 | 6.6 | 0.0 |

**Table G. The impact of high applied voltage for a short duration on MNV-1 recovery in the MEBS system.** Values are average of duplication experiments (n=2).

| **Voltage** | | **0V** | **100V** | | **150V** | |
| --- | --- | --- | --- | --- | --- | --- |
| **Duration** | | **10 min** | **5 min** | **10 min** | **5 min** | **10 min** |
| **% recovery** | sample chamber | 89.5 | 56.6 | 38.3 | 46.3 | 22.7 |
|  | collection chamber | 0.4 | 18.8 | 20.8 | 24.5 | 14.4 |
| **Standard deviation** | sample chamber | 0 | 11.6 | 5.1 | 2.3 | 2.3 |
|  | collection chamber | 0 | 0.2 | 4.7 | 7.2 | 4.7 |

**Table H. MNV-1 recovery using a horizontal electro-separation device without the addition of 652 membrane barriers for restriction or separation (n=1).**

| **Voltage** | | **0V** | **40V** | **40V** | **20V** |
| --- | --- | --- | --- | --- | --- |
| **Duration** | | **30 min** | **10 min** | **30 min** | **30 min** |
| **% recovery** | sample chamber | 50.1 | 0.0 | 0.0 | 0.0 |
|  | collection chamber | 48.9 | 0.0 | 0.0 | 0.0 |
